# Associations of healthy eating index-2015 with osteoporosis and low bone mass density in postmenopausal women: a population-based study from NHANES 2007–2018

**DOI:** 10.3389/fnut.2024.1388647

**Published:** 2024-04-17

**Authors:** Kai Wang, Jinyi Wu, Minggang Deng, Fengxi Tao, Qingwen Li, Xin Luo, Fang Xia

**Affiliations:** ^1^Department of Public Health, Wuhan Fourth Hospital, Wuhan, China; ^2^Department of Psychiatry, Wuhan Mental Health Center, Wuhan, China; ^3^Department of Psychiatry, Wuhan Hospital for Psychotherapy, Wuhan, China

**Keywords:** diet quality, HEI-2015, osteoporosis, bone mineral density, postmenopausal women

## Abstract

**Purpose:**

The current study aimed to explore the associations of diet quality assessed by healthy eating index-2015 (HEI-2015) with risks of osteoporosis and low bone mineral density (BMD) among American postmenopausal women aged 50 years and older.

**Methods:**

Postmenopausal women aged 50 years and older in the National Health and Nutrition Examination Survey from 2007 through 2018 were included in the final sample. Analysis of variance and Rao-Scott adjusted chi-square tests were used to compare the characteristics across tertiles of HEI-2015. Univariate and multivariate weighted logistic regression models were employed to explore the associations of HEI-2015 tertiles and continuous HEI-2015 with the risks of osteoporosis and low BMD. Nonlinear dose-response associations were evaluated using weighted restricted cubic spline analyses, and the contributions of various HEI-2015 components were assessed using weighted quantile sum regression models.

**Results:**

The final sample included 3,421 postmenopausal women aged 50 years and older representative for approximately 28.38 million non-institutionalized U.S. postmenopausal women. Osteoporosis prevalence decreased with HEI-2015 tertiles while the prevalence of low BMD showed no significant decrease. Compared with postmenopausal women in the first tertile of HEI-2015, those with the second (OR: 0.57, 95%CI: 0.38–0.84) and third (OR: 0.48, 95%CI: 0.29–0.78) HEI-2015 tertiles were associated with reduced osteoporosis risk after multivariate adjustments, but no significant association of HEI-2015 with the risk of BMD was identified. Furthermore, similar effects were confirmed in the sensitivity analyses and subgroup analyses and interaction effects. Moreover, significant nonlinear associations were observed between HEI-2015 with osteoporosis risk, and total vegetables, refined grains and greens and beans demonstrated the strongest protective effect among HEI-2015 components against osteoporosis.

**Conclusions:**

This study strongly suggests the significant negative associations of HEI-2015 with osteoporosis risk in American postmenopausal women. These findings highlight the importance of adherence to the dietary guidelines for Americans in reducing the risk of osteoporosis.

## Introduction

Osteoporosis, a systemic skeletal disease characterized by reduced bone mineral density (BMD) and degradation of bone microstructure, has emerged as a prominent public health concern with the global prevalence being 19.7% (1, 2). Consequently, it leads to increased bone fragility and susceptibility to fractures, resulting in approximately an anual cost of 17.9 billion dollars and 4 billion pounds in the USA and UK of osteoporosis-related fracture (3). According to the International Osteoporosis Federation, 10.2 and 43.4 million adults aged 50 years and older were estimated to have osteoporosis and low BMD in the United States in 2010, and the prevalence were 10.3% and 43.9% (4). These figures highlight the significant burden of osteoporosis and low BMD in the US population, particularly among older adults. Furthermore, the significant correlation between the prevalence of osteoporosis and increasing age were approved by numerous studies. Moreover, the global society is currently experiencing a rapid shift in its age structure, with populations becoming increasingly dominated by older individuals. As a consequence, the prevalence of osteoporosis is expected to rise predictably in the coming years, in which managements of osteoporosis is urgently need to address this public health issue effectively.

Postmenopausal women are particularly susceptible to low BMD and osteoporosis due to the combination of age and hormonal changes. Specifically, estrogen plays a crucial role in maintaining BMD and it decreases significantly during menopause. As a result, the prevalence of osteoporosis in postmenopausal women is notably elevated to be about 40% in Caucasia while it varied between 15% and 33% in Brazil, depending on the methodology employed and the use of bone densitometry data or self-reporting by participants (5, 6). Therefore, the management of postmenopausal osteoporosis is indeed a pressing need. While exercise and medications play important roles in its treatment, dietary implementation is also recognized as a valuable measure for preventing and managing osteoporosis (7–15).

In addition to calcium and vitamin D, multiple studies have evaluated the influence of dietary nutrients intake such as potassium, vitamin K, vitamin C and total protein intake on osteoporosis (16–25). Beyond individual nutrients intake, the overall dietary pattern and quality such as dietary total antioxidant capacity have gained attention as a comprehensive approach to nutrition (26). However, studies specifically focusing on diet pattern and quality in relation to osteoporosis are relatively limited, with much of the research centered around the benefits of the Mediterranean diet and dietary approaches to stop hypertension (DASH) (27–30). Furthermore, it is important to note that studies examining diet pattern and quality vary in terms of measurement methods and target populations, resulting in heterogeneity in the results across different studies.

The healthy eating index-2015 (HEI-2015), a measure to assess the degree of individual food intake align with Dietary Guidelines of Americans (DGA), is adopted in plenty of studies to reflect diet quality and it is of great construct validity, reliability, and criterion validity (31, 32). To the best of our knowledge, our study is the first to explore the correlation of diet quality assessed by HEI-2015 and osteoporosis risk in postmenopausal women. With data from 2007 to 2018 National Health and Nutrition Examination Survey (NHANES), we aimed to investigate the associations of HEI-2015 with the risks of low BMD and osteoporosis among postmenopausal women aged 50 years and older. By examining these associations, we can provide valuable insights into the role of diet quality in preventing osteoporosis and help develop comprehensive dietary guidelines for promoting bone health in postmenopausal women.

## Methods

### Study population

NHANES, conducted by the National Center for Health Statistics of the Centers for Disease Control and Prevention, is a consecutive and population-based study carried out every 2 years to evaluate the nutrition and health status of the U.S. non-institutionalized population. NHANES encompasses a wide range of data, including demographics, dietary, examination, laboratory and questionnaire data, providing detailed information about demographics characteristics, socioeconomic status, physiological measurements, biochemical indicators and standardized questionnaires about health in various aspects. To ensure the reliability and representativeness of the data, NHANES implements a complex, multistage, probability sampling design, as well as oversampling of specific subpopulations. Additionally, the compensation provided to participants helps to ensure the collection of reliable and high-quality data.

**Figure 1:**
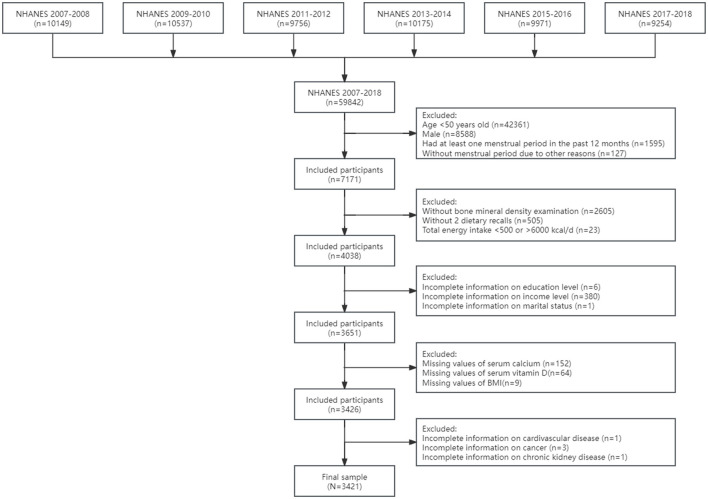
The flowchart of sample design. BMI, body mass index; NHANES, National Health and Nutrition Examination Survey.

In the current study, we included 7,171 postmenopausal women aged 50 years and older in 6 cycles from NHANES 2007–2008 to 2017–2018. Menopausal status was defined based on the self-reported reproductive health questionnaire. Postmenopausal women were limited to participants who answered “no” to the question “Have you had at least one menstrual period in the past 12 months?”and subsequently answered “hysterectomy” or “menopause/change of life” to the question “What is the reason that you have not had a period in the past 12 months?”. Details of the current study sampling, and exclusion criteria are described in Figure 1. A total of 2,605 adults without bone mineral density examination and 505 individuals without complete dietary recall data were excluded from the study, and participants whose total energy intake >6,000 or < 500 kcal per day (*n* = 23) were excluded to eliminate the influences of extreme individuals. Additionally, the study excluded adults without information on education (*n* = 6), family income (*n* = 380) and marital status (*n* = 1). After excluding participants with missing values of serum calcium (*n* = 152), vitamin D (*n* = 64) and body mass index (BMI) as well as without detailed information on cardiovascular disease (*n* = 1), cancer (*n* = 3) and chronic kidney disease (*n* = 1), the final sample included 3,421 postmenopausal women with complete data for analysis in the study.

### Outcome ascertainment

Participants underwent BMD examinations by dual-energy X-ray absorptiometry in mobile examination center by trained and certified radiology technologists, in which pregnant females, participants with self-reported history of radiographic contrast material in the past 7 days or with measured weight over 450 pounds met the exclusion criteria from the DXA examination. Detailed descriptions are provided in the DXA examination protocol documented in the Body Composition Procedures Manual. Low BMD and osteoporosis were defined on the basis of the total femur (TF), femoral neck (FN), and lumbar spine (LS) BMD measurements used in previous studies (33). The mean BMD values of female participants aged between 20 and 29 years old was used as the reference values. Individuals with any BMD value < 2.5 standard deviations below the reference value were considered as osteoporosis, while individuals with any BMD value < 1 standard deviations below the reference value were considered was low BMD (34, 35). Further details of female participants aged between 20 and 29 years old are listed in Supplementary Table 1.

### Exposures

The HEI-2015 is established to assess diet quality, specifically the degree to which a set of foods aligns with the 2015–2020 DGA. HEI-2015 has been developed from the HEI-2010 by replacing empty calories with saturated fat and added sugar, with the result being 13 components (31). Although the most recent edition of DGA (2020–2025) has been published and corresponding HEI-2020 has been developed, the 13 components and scoring standards of the HEI-2020 fully align with the HEI-2015 and it was renamed just to clarify the consistency of 2020–2025 DGA (36). HEI-2015 is a density-based index specifically based on dietary nutrients intake per 1,000 kcal rather than absolute amount, and the total score range from 0 to 100 in which higher score indicate higher adherence to 2015–2020 DGA and higher diet quality. Nutrients intakes and alcohol consumption were calculated with the mean of two 24-h dietary recalls and then HEI-2015 were obtained by corresponding scoring standards, in which a face-to-face interview in the first day and a follow-up interview 3–10 days later by telephone were conducted. For subsequent statistical analyses, HEI-2015 was categorized into three groups with tertiles and the lowest tertile was set as the reference group.

### Assessment of covariates

Various demographic variables were taken into consideration in current study, including age group (middle-aged, 50–64 years; older, ≥65 years), race (non-Hispanic White, non-Hispanic Black, Mexican Americans, and other races), education level (less than high school degree, high school degree, more than high school degree), family income level (measured as the ratio of family income to poverty (PIR), low family income: PIR ≤ 1.3, medium family income: 1.3 < PIR < 3.5, high family income: PIR ≥ 3.5), marital status (married or living with partner; divorced, separated, or widowed; never married). In additional, lifestyle factors such as BMI (normal or low body weight: < 25, overweight: 25–29.9, obese: ≥30), serum cotinine levels (low: < 1, medium: 1–10, high: ≥10), alcohol consumption (nondrinker: = 0, moderate drinker: 0–30 g/day for men and 0–15 g/day for women, heavy drinker: ≥ 30 g/day for men and ≥ 15 g/day for women), and leisure time physical activity (LTPA, calculated as twice the duration of vigorous physical activity plus the duration of moderate physical activity) were adjusted (37–40). Furthermore, total energy intake (expressed as kilocalorie) was adjusted for as the components of HEI-2015 were density-based rather than absolute dietary nutrients intake, and serum calcium and vitamin D were also taken into account. A series of chronic non-communicable disease including hypertension, cardiovascular diseases (CVD), diabetes, chronic kidney disease (CKD) and cancer were included on account of the associations with BMD. Hypertension was defined as average systolic pressure ≥ 140 mm Hg and/or diastolic pressure ≥ 90 mm Hg in 3 tests or self-reported hypertension. CVD was defined as self-reported diagnosis of congestive heart failure, coronary heart disease, angina, myocardial infarction or stroke by a professional doctor. Diabetes was defined as fasting plasma glucose ≥ 7.0 mmol/L, 2-h plasma glucose ≥ 11.0 mmol/L, hemoglobin A1c ≥ 6.5% or self-reported diabetes by a professional doctor. CKD was defined as an estimated glomerular filtration rate < 75 with the CKD-Epidemiology Collaboration (EPI) equation (41). Cancer was defined as self-reported cancer or a malignancy by a professional doctor or other health professional.

### Statistical analysis

According to analytic guidelines in NHANES, dietary two-day sample weight, clustering, and stratification were taken into account. Moreover, dietary two-day sample weight divided by 6 was utilized to make the final sample representative of the national non-institutionalized population as the data of 6 consecutive cycles were combined in our analyses.

Statistical descriptions were presented by continuous variables expressed as weighted means (standard deviations), and categorical variables expressed with numbers (weighted percentages). Analyses of variance and Rao-Scott adjusted chi-square tests were used to compare the characteristics between adults across different HEI-2015 tertiles. Univariate and multivariate weighted logistic regression models were employed to explore the associations of HEI-2015 with low BMD and osteoporosis in the general population, in which model 1 was unadjusted, model 2 was adjusted for demographics variables (Age group, race, education level, income level, marital status) while model 3 was the fully adjusted model additionally adjusted for BMI status, serum cotinine level, alcohol consumption, LTPA, serum calcium and vitamin D and comorbidity (hypertension, CVD, diabetes, CKD and cancer) based on model 2. Moreover, trend tests (p for trend) were performed by entering the tertile-categorical HEI-2015 as a continuous variable and rerunning the corresponding regression models. Three sensitivity analyses were further conducted to validate the robustness of our results: (1) HEI-2015 was categorized into quartiles but not tertiles; (2) the definition of low BMD and osteoporosis was revised to be based on femoral neck and lumber spine BMD while total femur BMD was not considered; (3) we excluded participants who had previously taken anti-osteoporotic drugs to eliminate the influence. Stratified analyses were conducted to investigate whether the associations differ by demographic variables (age group, race, education level, income level and marital status) and interaction effects were tested. Weighted restricted cubic splines (RCS) were utilized to examine the nonlinear correlations of HEI-2015 with low BMD and osteoporosis, with three knots located at the 25th, 50th, and 75th percentiles of the distributions. Weighted quantile sum (WQS) regression models were employed to assess the contributions of various components to reducing the osteoporosis risk. The individual weight for each component in the HEI-2015 was estimated using quartiles (q=4) through bootstrap sampling (*n* = 100), where the data were randomly split into the training set (90%) and the validation set (10%).

All other statistical analyses were performed in Stata software (version 17.0, StataCorp LLC) except for analyses of variance and WQS in R software (version 4.2.2). All statistical tests were two-sided, and significance was considered at α = 0.05.

## Results

### Characteristics

Characteristics of postmenopausal women grouped by tertiles of HEI-2015 were presented in Table 1. The final sample included 3,421 participants representative for 28.38 million non-institutionalized postmenopausal women (mean [SD] HEI-2015, 57.72 [13.00]; mean [SD] age, 62.63 [8.85]; 1,731 [weighted 75.5%] non-Hispanic White). Meanwhile, the prevalence of low BMD and osteoporosis are 65.8% and 10.2%, respectively.

**Table 1:** The characteristics by tertiles of the HEI-2015.

**Characteristics**	**Tertile 1 ( ≤ 51.82) (*n* = 1,175)**	**Tertile 2 (51.82–63.45) (*n* = 1,072)**	**Tertile 3 (>63.45) (*n* = 1,174)**	**Overall (*N* = 3,421)**	***P* value**
**Age group** (n/%)					0.0846
Middle-aged (50–64 y)	729 (67.7%)	591 (62.8%)	619 (60.4%)	1,939 (63.6%)	
Older (≥65 y)	446 (32.3%)	481 (37.2%)	555 (39.6%)	1,482 (36.4%)	
**Race** (n/%)					0.0041
Non-Hispanic White	600 (73.4%)	563 (78.4%)	568 (74.6%)	1,731 (75.5%)	
Non-Hispanic Black	275 (11.7%)	199 (7.9%)	190 (7.5%)	664 (9.0%)	
Mexican Americans	147 (5.1%)	126 (4.5%)	142 (4.5%)	415 (4.7%)	
Other races	153 (9.7%)	184 (9.2%)	274 (13.5%)	611 (10.8%)	
**Education level** (n/%)					< 0.0001
< High school	292 (16.4%)	235 (13.0%)	229 (11.9%)	756 (13.8%)	
High school	361 (34.8%)	257 (24.8%)	250 (18.9%)	868 (26.2%)	
>High school	522 (48.8 %)	580 (62.1%)	695 (69.2%)	1,797 (60.0%)	
**Family income level** (n/%)					0.0005
Low	379 (21.7%)	276 (15.5%)	279 (14.2%)	934 (17.1%)	
Medium	488 (38.8%)	427 (33.1%)	439 (34.4%)	1,354 (35.4%)	
High	308 (39.5%)	369 (51.5%)	456 (51.4%)	1,133 (47.5%)	
**Marital Status** (n/%)					0.0010
Married or living with partner	558 (53.5%)	601 (66.8%)	631 (62.3%)	1,790 (60.9%)	
Divorced, separated, or widowed	533 (40.7%)	401 (29.6%)	472 (32.4%)	1,406 (34.2%)	
Never married	84 (5.8%)	70 (3.6%)	71 (5.3%)	225 (4.9%)	
**BMI status** (n/%)					< 0.0001
Normal or low body weight	251 (23.6%)	286 (26.4%)	358 (36.6%)	895 (28.9%)	
Overweight	350 (27.8%)	367 (35.1%)	425 (33.7%)	1,142 (32.2%)	
Obese	574 (48.6%)	419 (38.5%)	391 (29.7%)	1,384 (38.9%)	
**Serum cotinine** (n/%)					< 0.0001
Low (< 1)	816 (70.9%)	894 (81.7%)	1,079 (91.4%)	2,789 (81.3%)	
Medium (1–10)	34 (1.9%)	20 (2.3%)	18 (1.4%)	72 (1.8%)	
High (≥10)	325 (27.2%)	158 (16.0%)	77 (7.3%)	560 (16.8%)	
**Alcohol consumption** (n/%)					0.0003
Nondrinker	971 (80.0%)	815 (72.1%)	880 (65.3%)	2,666 (72.5%)	
Moderate drinker	115 (10.3%)	128 (12.0%)	166 (18.2%)	409 (13.5%)	
Heavy drinker	89 (9.7%)	129 (15.9%)	128 (16.6%)	346 (14.1%)	
**Hypertension** (n/%)	860 (67.7%)	740 (61.6%)	775 (59.3%)	2,375 (62.9%)	0.0720
**CVD** (n/%)	198 (14.3%)	159 (14.6%)	128 (9.5%)	485 (12.8%)	0.0230
**Diabetes** (n/%)	310 (21.1%)	280 (20.0%)	265 (16.6%)	855 (19.2%)	0.1659
**CKD** (n/%)	367 (29.4%)	341 (31.9%)	381 (32.8%)	1,089 (31.3%)	0.5879
**Cancer** (n/%)	180 (15.4%)	183 (18.3%)	185 (18.0%)	548 (17.2%)	0.3957
**Low BMD** (n/%)	770 (65.8%)	734 (65.8%)	786 (65.9%)	2,290 (65.8%)	0.9984
**Osteoporosis** (n/%)	138 (13.5%)	125 (8.7%)	120 (8.4%)	383 (10.2%)	0.0176
**Serum calcium (mg/dL)**, Mean (SD)	9.44 (0.39)	9.47 (0.36)	9.48 (0.40)	9.46 (0.38)	0.149
**Serum vitamin D (nmol/L)**, Mean (SD)	75.28 (34.20)	81.12 (32.32)	85.91 (34.82)	80.77 (34.07)	< 0.001
**Total energy intake (kcal)**, Mean (SD)	1,744.45 (621.88)	1,771.99 (595.99)	1,673.97 (527.78)	1,730.10 (584.48)	0.086
**LTPA (min/wk)**, Mean (SD)	82.56 (190.97)	140.71 (252.49)	167.80 (272.39)	130.40 (243.70)	< 0.001

BMD, bone mineral density; CKD, chronic kidney disease; CVD, cardiovascular disease; LTPA, leisure time physical activity.

In comparison to the lowest HEI-2015 tertile, adults in higher tertiles were more likely to be non-Hispanic White, married or living with partner, normal or low body weight, and less likely to be non-drinker and comorbid with CVD and osteoporosis. Moreover, adults in higher tertiles had higher education level, family income level, LTPA, and serum vitamin D and lower serum cotinine. Nevertheless, no significant differences in low BMD prevalence across groups of HEI-2015 tertiles were observed.

### Associations of HEI-2015 with low BMD and osteoporosis

As described in Table 2, stepped weighted logistic regression models revealed the negative associations of continuous HEI-2015 and HEI-2015 tertiles with osteoporosis risk in 3 models, but no significant relationship between HEI-2015 and low BMD was observed, neither continuous HEI-2015 nor HEI-2015 tertiles. Compared with the lowest HEI-2015 tertile, the second (OR: 0.57, 95%CI: 0.38–0.84) and the third (OR: 0.48, 95%CI: 0.29–0.78) tertiles were associated with lower risks of osteoporosis in the fully adjusted model. Additionally, there were significant trends observed across the HEI-2015 tertiles. Moreover, all three sensitivity analyses demonstrated similar correlations and trends as shown in Table 3, suggesting the strong and consistent associations between continuous HEI-2015, HEI-2015 tertiles, and the risk of osteoporosis.

**Table 2:** The associations of HEI-2015 with low BMD and osteoporosis.

**HEI-2015**	**Low BMD**			**Osteoporosis**		
	**Model 1**	**Model 2**	**Model 3**	**Model 1**	**Model 2**	**Model 3**
Continuous	1.002 (0.994,1.011)	1.002 (0.993,1.011)	0.998 (0.988,1.007)	0.982 (0.968,0.996)	0.981 (0.966,0.996)	0.977 (0.961,0.992)
T1	ref	ref	ref	ref	ref	ref
T2	1.00 (0.75,1.34)	0.98 (0.73,1.33)	0.97 (0.71,1.33)	0.61 (0.42,0.90)	0.61 (0.42,0.89)	0.57 (0.38,0.84)
T3	1.00 (0.77,1.32)	0.96 (0.72,1.27)	0.86 (0.64,1.15)	0.59 (0.37,0.93)	0.54 (0.34,0.86)	0.48 (0.29,0.78)
*P* trend	0.972	0.762	0.298	0.021	0.010	0.004

BMD, bone mineral density.

Model 1 was unadjusted.

Model 2 was adjusted for demographics data.

Model 3 was the fully adjusted model adjusted for demographics data, BMI status, smoking status, alcohol consumption, LTPA, total energy intake, serum calcium and vitamin D, and comorbidity.

**Table 3:** The sensitivity analyses of associations of HEI-2015 with low BMD and osteoporosis.

**HEI-2015**	**Low BMD**			**Osteoporosis**		
	**Model 1**	**Model 2**	**Model 3**	**Model 1**	**Model 2**	**Model 3**
**Sensitivity analysis 1 (HEI-2015 categorized into quartiles)**						
Continuous	1.002 (0.994,1.011)	1.002 (0.993,1.011)	0.998 (0.988,1.007)	0.982 (0.968,0.996)	0.981 (0.966,0.996)	0.977 (0.961,0.992)
Q1	ref	ref	ref	ref	ref	ref
Q2	1.03 (0.75,1.42)	1.09 (0.77,1.53)	1.09 (0.76,1.57)	0.85 (0.56,1.28)	0.89 (0.59,1.35)	0.88 (0.57,1.37)
Q3	1.11 (0.75,1.65)	1.10 (0.74,1.63)	1.01 (0.67,1.52)	0.69 (0.44,1.10)	0.68 (0.40,1.15)	0.61 (0.38,0.99)
Q4	1.04 (0.76,1.43)	1.06 (0.77,1.45)	0.93 (0.66,1.30)	0.61 (0.37,1.01)	0.61 (0.37,1.01)	0.54 (0.31,0.92)
*P* trend	0.712	0.754	0.577	0.047	0.047	0.015
**Sensitivity analysis 2 (The definition of low BMD and osteoporosis based on femoral neck and lumber spine)**						
Continuous	1.003 (0.994,1.011)	1.002 (0.993,1.011)	0.997 (0.988,1.007)	0.978 (0.964,0.992)	0.976 (0.961,0.992)	0.971 (0.956,0.987)
T1	ref	ref	ref	ref	ref	ref
T2	0.99 (0.74,1.33)	0.98 (0.72,1.32)	0.96 (0.71,1.31)	0.61 (0.41,0.91)	0.61 (0.41,0.90)	0.56 (0.37,0.84)
T3	1.01 (0.76,1.35)	0.96 (0.71,1.29)	0.85 (0.63,1.16)	0.41 (0.31,0.83)	0.47 (0.28,0.78)	0.41 (0.24,0.68)
P trend	0.928	0.780	0.302	0.006	0.003	0.001
**Sensitivity analysis 3 (Excluded participants who had previously taken anti-osteoporotic drugs)**						
Continuous	1.001 (0.992,1.010)	1.001 (0.991,1.011)	0.996 (0.986,1.006)	0.986 (0.972,0.999)	0.986 (0.972,0.999)	0.981 (0.966,0.996)
T1	ref	ref	ref	ref	ref	ref
T2	0.94 (0.68,1.28)	0.93 (0.68,1.29)	0.91 (0.65,1.27)	0.62 (0.41,0.91)	0.63 (0.42,0.95)	0.58 (0.38,0.87)
T3	0.94 (0.71,1.24)	0.90 (0.67,1.21)	0.80 (0.58,1.09)	0.60 (0.37,0.96)	0.56 (0.35,0.90)	0.49 (0.30,0.80)
*P* trend	0.663	0.483	0.145	0.032	0.019	0.006

BMD, bone mineral density.

Model 1 was unadjusted.

Model 2 was adjusted for demographics data.

Model 3 was the fully adjusted model adjusted for demographics data, BMI status, smoking status, alcohol consumption, LTPA, total energy intake, serum calcium and vitamin D, and comorbidity.

### Subgroup analyses and interaction effects of the associations of HEI-2015 with low BMD and osteoporosis

Tables 4, 5 exhibit the associations between HEI-2015 and low BMD with osteoporosis in demographic subpopulations in the fully adjusted models. The relationship between HEI-2015 and the risk of low BMD was not observed in all subpopulations except for older adults in which the trends were also observed. Furthermore, the study did not identify any interactions between demographic variables and HEI-2015 in relation to the risk of low BMD. Nevertheless, the correlations between HEI-2015 and the risk of osteoporosis were identified in various subgroups including all age groups, non-Hispanic White participants, individuals with less than and more than a high school education, high family income level and divorced, separated, or widowed postmenopausal women. Moreover, the trends were also identified to be significant. In addition, significant interaction effects of income (*P* = 0.0210) were shown, suggesting that the correlation was only identified to be significant in high family income subgroups.

**Table 4:** The relationship between HEI-2015 and low BMD in demographic subgroups.

**Characteristics**	**T1**	**T2**	**T3**	***P* for interaction**	***P* for trend**
**Age group** (n/%)				0.5013	
Middle-aged adults (50–64 y)	Ref	1.04 (0.73,1.49)	1.00 (0.71,1.40)		0.977
Older adults (≥65 y)	Ref	0.78 (0.45,1.37)	0.56 (0.32,0.98)		0.038
**Race** (n/%)				0.3546	
Non-Hispanic White	Ref	0.94 (0.62,1.41)	0.83 (0.56,1.41)		0.354
Non-Hispanic Black	Ref	0.80 (0.48,1.33)	1.01 (0.59,1.75)		0.973
Mexican Americans	Ref	0.80 (0.38,1.67)	1.00 (0.51,1.94)		0.923
Other races	Ref	1.61 (0.69,3.73)	0.56 (0.29,1.08)		0.064
**Education level** (n/%)				0.9315	
< High school	Ref	0.80 (0.43,1.46)	0.94 (0.50,1.77)		0.817
High school	Ref	1.16 (0.63,2.13)	0.98 (0.53,1.80)		0.955
>High school	Ref	0.94 (0.59,1.49)	0.80 (0.51,1.26)		0.313
**Family income level** (n/%)				0.7785	
Low	Ref	0.86 (0.47,1.57)	0.71 (0.44,1.15)		0.162
Medium	Ref	0.96 (0.55,1.67)	1.05 (0.62,1.79)		0.863
High	Ref	0.93 (0.56,1.57)	0.75 (0.47,1.20)		0.204
**Marital status** (n/%)				0.5044	
Married or living with partner	Ref	0.89 (0.57,1.39)	0.79 (0.53,1.17)		0.224
Divorced, separated, or widowed	Ref	0.99 (0.55,1.77)	1.01 (0.60,1.70)		0.976
Never married	Ref	1.33 (0.42,4.17)	0.53 (90.15,1.91)		0.369

All models were fully adjusted for demographics data, BMI status, smoking status, alcohol consumption, LTPA, total energy intake, serum calcium and vitamin D, and comorbidity.

**Table 5:** The relationship between HEI-2015 and osteoporosis in demographic subgroups.

**Characteristics**	**T1**	**T2**	**T3**	***P* for interaction**	***P* for trend**
**Age group** (n/%)				0.3447	
Middle-aged adults (50–64 y)	Ref	0.57 (0.29,1.13)	0.35 (0.14,0.83)		0.020
Older adults (≥65 y)	Ref	0.56 (0.34,0.91)	0.54 (0.32,0.93)		0.033
**Race** (n/%)				0.7028	
Non-Hispanic White	Ref	0.54 (0.33,0.90)	0.42 (0.23,0.77)		0.006
Non-Hispanic Black	Ref	0.87 (0.26,2.93)	0.85 (0.32,2.30)		0.753
Mexican Americans	Ref	0.54 (0.26,1.13)	0.71 (0.25,2.00)		0.507
Other races	Ref	0.82 (0.25,2.05)	0.56 (0.23,1.39)		0.207
**Education level** (n/%)				0.1092	
< High school	Ref	0.79 (0.40,1.58)	0.30 (0.11,0.82)		0.015
High school	Ref	1.04 (0.50,2.16)	0.98 (0.46,2.08)		0.971
>High school	Ref	0.40 (0.21,0.77)	0.38 (0.19,0.76)		0.011
**Family income level** (n/%)				0.0210	
Low	Ref	0.98 (0.52,1.85)	0.71 (0.36,1.43)		0.365
Medium	Ref	0.94 (0.54,1.64)	0.63 (0.31,1.27)		0.188
High	Ref	0.22 (0.10,0.48)	0.26 (0.13,0.53)		0.001
**Marital status** (n/%)				0.7524	
Married or living with partner	Ref	0.61 (0.35,1.08)	0.59 (0.33,1.06)		0.081
Divorced, separated, or widowed	Ref	0.52 (0.28,0.99)	0.42 (0.21,0.83)		0.013
Never married	Ref	1.02 (0.30,1.47)	0.28 (0.05,1.65)		0.155

All models were fully adjusted for demographics data, BMI status, smoking status, alcohol consumption, LTPA, total energy intake, serum calcium and vitamin D, and comorbidity.

### Nonlinear associations of HEI-2015 with low BMD and osteoporosis

**Figure 2:**
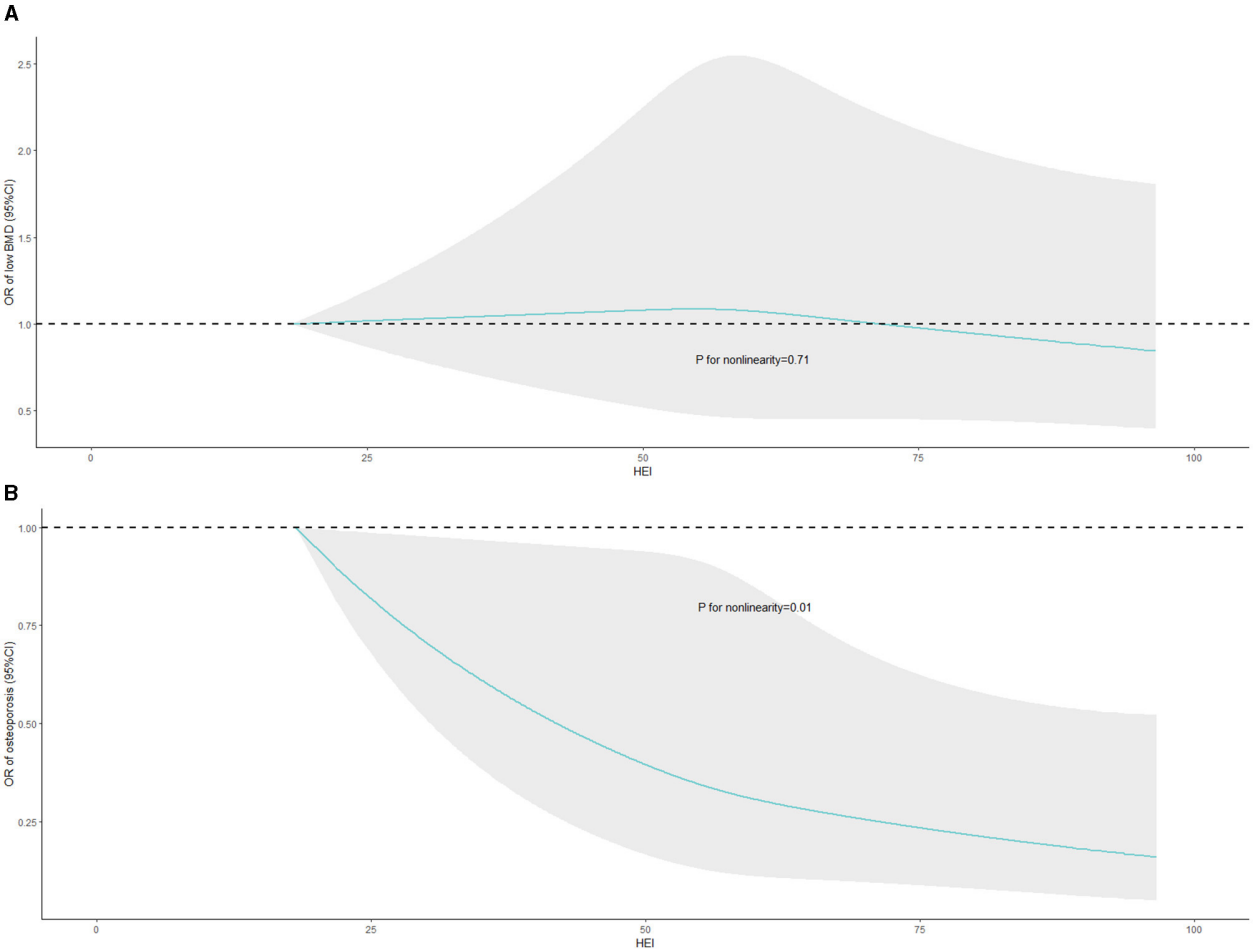
Nonlinear associations of HEI-2015 with the risks of low BMD and osteoporosis with weighted RCS. BMD, bone mineral density; HEI-2015, healthy eating index-2015; RCS, restricted cubic splines. The fully adjusted model was exclusively employed in the WQS model, encompassing adjustments for demographics data, BMI status, smoking status, alcohol consumption, LTPA, total energy intake, serum calcium and vitamin D levels, and comorbidities.

Weighted RCS were conducted to assess the nonlinear associations of HEI-2015 with the risks of low BMD and osteoporosis in the fully adjusted model, the results of which were displayed in Figure 2. Significant negative non-linear association of HEI-2015 and osteoporosis risk (*P* = 0.0112) was observed in Figure 2B while no significant relationship between HEI-2015 and the risk of low BMD (*P* = 0.7130) was demonstrated in Figure 2A. The results suggest that as HEI-2015 increase, osteoporosis risk decrease in a nonlinear manner.

### Mixed effect of HEI-2015 on osteoporosis

**Figure 3:**
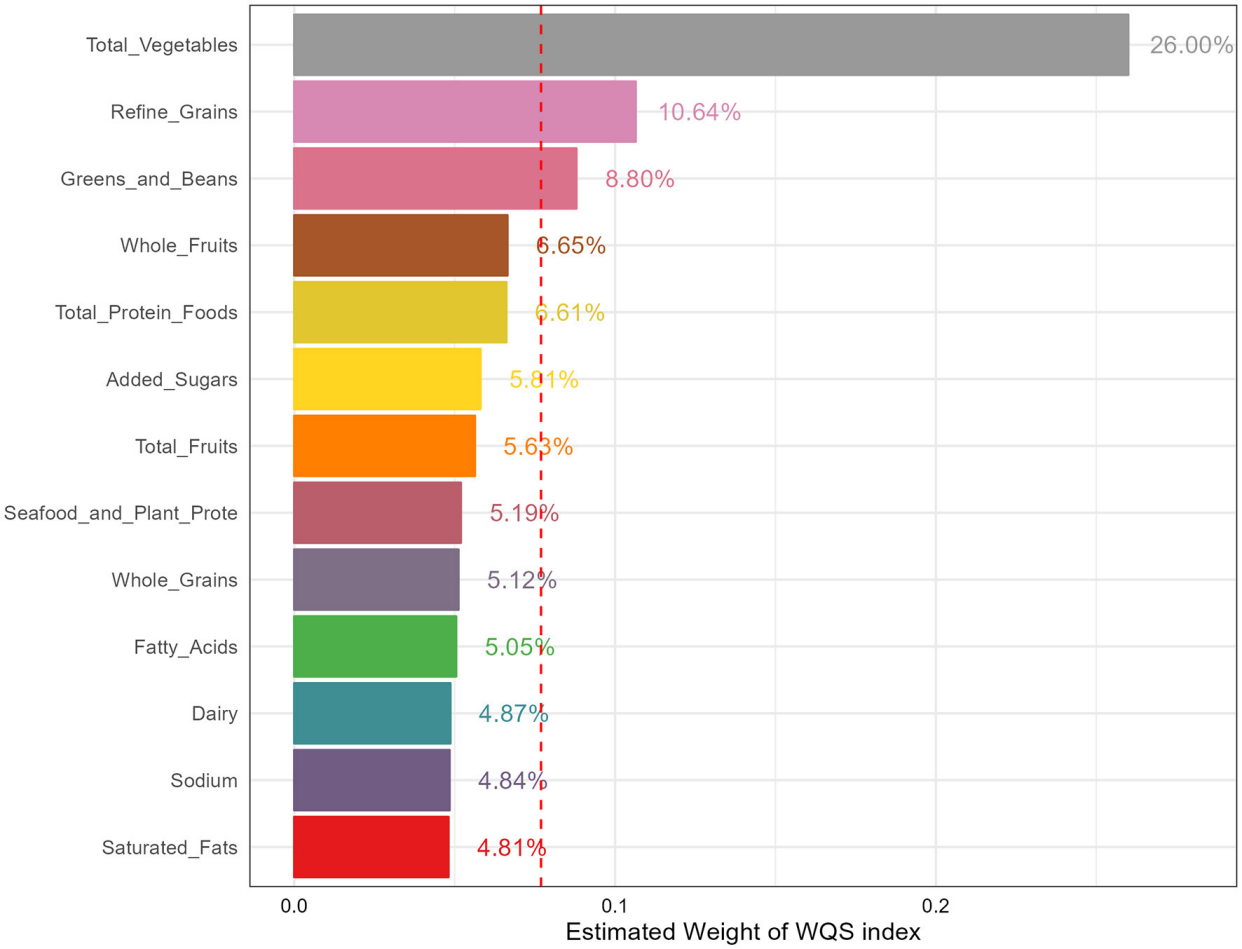
The estimated weight of various components in HEI-2015. HEI-2015, healthy eating index-2015; WQS, weighted quantile sum.

WQS regression models were employed and the results were shown in Figure 3 to assess the impact of various components on reducing the risk of osteoporosis. In the fully adjusted model, the WQS index of HEI-2015 (OR: 0.16, 95%CI: 0.06–0.45) demonstrated the significant association with decreased risk of osteoporosis. Specifically, total vegetables (26.00%), refined grains (10.64%) and greens and beans (8.80%) were identified as the most weighted components, indicating that these three components contributed the most to reducing the osteoporosis risk.

The fully adjusted model was exclusively employed in the WQS model, encompassing adjustments for demographics data, BMI status, smoking status, alcohol consumption, LTPA, total energy intake, serum calcium and vitamin D levels, and comorbidities.

## Discussions

Based on data from 6 cycles of the large cross-sectional survey, we found that diet quality assessed by HEI-2015 was negatively associated with osteoporosis risk in postmenopausal women aged 50 years and older while no significant association between HEI-2015 and risk of low BMD was identified. Moreover, these results were consistent across three sensitivity analyses, suggesting the robustness of our results. In addition, subgroup analyses and interaction effects demonstrated the stability of the associations in various demographic subgroups, with a particularly pronounced effect observed in participants with high family income. Furthermore, weighted RCS indicated the existence of non-linear association of HEI-2015 and osteoporosis risk, suggesting the osteoporosis risk decrease with HEI-2015 in a non-linear manner. Finally, we determined the contributions of different components of HEI-2015 to reducing the risk of osteoporosis with WQS. We found that total vegetables, refined grains, and greens and beans had the highest percentages of contribution, amounting to 26.00%, 10.64%, and 8.80% respectively. These findings suggest that focusing on these specific components of the diet may be particularly beneficial in reducing the risk of osteoporosis.

Apart from nutrients intake and food groups, recent studies have emphasized the importance of evaluating overall dietary patterns on osteoporosis and BMD and found the beneficial effect, since it takes into account the overall combination and balance of foods consumed and nutrients intake, and have a broader and more comprehensive approach to nutrition. For instance, a Southern Spain study found the significant linear trends between the Mediterranean diet score and BMD, indicating the benefits of a varied diet based on Mediterranean diet patterns may be beneficial in the prevention of osteoporosis in postmenopausal women (27). Another study involving 418 healthy volunteers concluded that higher adherence to the Mediterranean diet was associated with higher T-score indicating better bone health status (28). Furthermore, a meta-analysis consisting of 6 cohorts, 6 cross-sectional and 1 case-control studies identified the higher BMD in participants with higher adherence to the Mediterranean diet (29). Similarly, higher DASH score was found to be associated with lower risk of osteoporosis at lumbar spine, but no significant relationship between DASH score and risk of osteoporosis at femoral neck was observed (30). Moreover, the Boston Puerto Rican osteoporosis study found that DASH was more positively associated with BMD than alternative HEI or Mediterranean diet score in postmenopausal women without estrogen (42).

HEI-2015, a measure to assess the degree of individual food intake align with DGA, was adopted in the current study to reflect diet quality. A similar study focusing on middle-aged and older Americans has evaluated the association between HEI-2015 total and component food scores with osteoporosis, and they found the significant negative association, similar to our study (43). In comparison with that study, we further extended the analyses and explored the associations in postmenopausal women and adjusted for various confounding factors including demographics variables, lifestyle factors, dietary and serum nutrition status, and comorbidities. In addition, we conducted sensitivity analyses, subgroup analyses, interaction effect, RCS and WQS models. Considering the robustness of the results, the dose-response relationships, and the contributions of various components, our study provides important insights into the associations of HEI-2015 and osteoporosis in postmenopausal women.

The results of RCS models revealed the significant association of HEI-2015 and osteoporosis risk in a non-linear manner. Specifically, the dose-response curves indicated a steep relationship between HEI-2015 and osteoporosis risk when the HEI-2015 score was relatively low. However, as the HEI-2015 score increased to higher levels, the relationship tended to become smoother. It cannot be denied that the improvements in diet quality may have a substantial impact on reducing the risk of osteoporosis especially HEI-2015 is at a poor or low level, and this finding suggests that improving diet quality from a poor or low level to a moderate level may have a more pronounced effect on reducing the risk of osteoporosis.

In addition, WQS displayed that total vegetables, refined grains, and greens and beans contributed the most on reducing the osteoporosis risk. Higher vegetables intake was found to be associated with lower osteoporosis risk in a cross-sectional study, and a meta-analysis concluded that higher vegetable-based diet intake was related with reduced osteoporosis risk (44). However, the results in a meta-analysis displayed the significant negative association of vegetable intake and postmenopausal women in case-control studies but not in cross-sectional studies, indicating the heterogeneity of results and encouraging more high-quality studies such as randomized controlled trials to explore the relationships (45). Furthermore, a two-sample Mendelian randomization study found the causal relationship between servings of raw vegetables per day and osteoporosis, providing strong literature evidence (46). The protective effect of vegetables against osteoporosis may be attributed to their rich content of vitamins and minerals like vitamin C, which have been described earlier to be negatively correlated with osteoporosis.

In addition, the similar article also found that the negative association of beans consumption and osteoporosis risk, and a study involving 1,433 Korean postmenopausal women also found the preventive effect of higher beans intake on osteoporosis (47). Furthermore, the experiment in a rat model of osteoporosis found that consumption of yellow and black soybeans, and sword beans had a definite protective effect on inhibiting bone turnover and preventing bone resorption, thus leading to less bone loss and higher BMD (48). Specifically, researchers speculated that isoflavones and phytochemicals in these beans build the defense against osteoporosis. For instance, soy isoflavones as phytoestrogens exerted estrogen-like effect and were believed to decrease bone resorption marker urine deoxypyridinoline, inhibiting bone resorption and increasing lumbar spine BMD (49, 50). Meanwhile, beans are a good source of plant protein and proteins intake is positively associated with BMD and negatively related with osteoporosis regardless of protein source (51–53). Our results additionally demonstrated the beneficial effect of higher refined-grains score and lower refined-grains intake, and this may be attributed to the fact that refined grains are known to contain fewer vitamins, minerals, and phytosterols that are important for bone health and protection against osteoporosis (54). Furthermore, refined grains were found to decrease BMD by modulating osteoprotegerin and receptor activator of nuclear factor kappa B (NF-κB) in male rats (55).

Apart from the direct positive effect of estrogen deficiency on postmenopausal osteoporosis, oxidative stress, inflammation and immune cell alterations have been acknowledged to contribute to the pathogenesis of postmenopausal osteoporosis, in which healthy diet may play an important role in attenuating the development. Excessive reactive oxygen species production due to estrogen insufficiency not only disrupts the formation and functionality of osteoblasts, but also negatively affects their activity, viability, proliferation, and apoptosis (56–60). This leads to a reduction in osteoblastic number and functionality with consequent beginning and development of osteoporotic processes, resulting in the altered bone architecture and bone loss that characterize osteoporosis. Postmenopausal women often exhibit a chronic low-grade inflammatory state with changes in cytokine expression and immune cell profile (61). Specifically, Estrogen deficiency activates the nucleotide-binding oligomerization domainlike receptor family pyrin domain-containing 3 (NLRP3) inflammasome expressed in osteoblasts and involved in immune innate response and inflammation, the abnormal activation of which plays an important role in the development of osteoporosis (62). Additionally, tissue necrosis factors α (TNF-α) was found to promote osteoblast apoptosis and indirectly stimulate osteoclastogenesis via B cell-produced receptor-activator of NF-κB ligand (RANKL), leading to bone loss during postmenopausal osteoporosis (63, 64). Interestingly, the gut-bone axis has emerged as a novel approach for the prevention and treatment of postmenopausal osteoporosis (65). Beneficial gut microbiota stimulates bone formation and inhibit bone resorption, making probiotic treatment a potential avenue for managing postmenopausal osteoporosis (66, 67). For instance, Prevotella was suggested to serve as a therapeutic agent or target for osteoporosis treatment since the proportion of Prevotella was identified lower in postmenopausal osteoporosis patients, and transplantation of Prevotella into ovariectomized mice helps in preventing bone loss (68).

The major strength of this study is the use of a large, nationally representative U.S. survey and the combination of data in 6 cycles, increasing the sample size and enhacing the generalizability of our results. Further, the adoptions of sensitivity analyses, subgroup analyses and interaction effects enhance the robustness and credibility of our results. Finally, weighted RCS and WQS models provided a more intuitive and comprehensive understanding of the dose-response relationships and contributions of various components. Nevertheless, it is undeniable that our study also has some limitations. Firstly, no causality but only associations could be inferred from this study as a result of the nature of cross-sectional studies. Secondly, some covariates were based on self-report but not medical records or medication, which may introduce potential bias and affect the reliability of the data. Finally, the effect of estrogen as the crucial determinant was unavailable in the participants, which leave out the effect of sex hormones.

## Conclusions

Among a nationally representative sample of U.S. postmenopausal women, we found the robust and negative associations of diet quality assessed by HEI-2015 and osteoporosis risk, but no significant association of low BMD was identified. Furthermore, the non-linear dose-response relationships remained stable in various sensitivity analyses and demographic subgroups, in which total vegetables, refined grains, and greens and beans contributed the most. By highlighting the relationships, we aim to emphasize the importance of adherence to dietary guidelines for Americans that can help reduce the osteoporosis risk.

## Data availability statement

The raw data supporting the conclusions of this article will be made available by the authors, without undue reservation.

## Author contributions

KW: Writing—original draft, Visualization, Validation, Supervision, Software, Methodology, Formal analysis, Data curation. JW: Writing—review & editing, Methodology. MD: Writing—review & editing, Methodology. FT: Writing—review & editing. QL: Writing—review & editing. XL: Writing—review & editing. FX: Writing—review & editing, Project administration.
