# Supplementary material for: Associations of healthy eating index-2015 with osteoporosis and low bone mass density in postmenopausal women: a population-based study from NHANES 2007–2018
Source: Front Nutr. 2024 Apr 17;11:1388647. doi: 10.3389/fnut.2024.1388647 (PMC11061362; doi:10.3389/fnut.2024.1388647)
Supplement: Supplementary file 1 [file Data_Sheet_1.docx]

Table S1 The bone mineral density of female adults aged between 20 and 29 years old

|  | Total Femur | Femoral Neck | Lumbar Spine |
| --- | --- | --- | --- |
| N | 316 | 316 | 459 |
| Mean | 0.972 | 0.885 | 1.057 |
| SD | 0.119 | 0.121 | 0.116 |

Figure S1 The receiver operating characteristic curve of the WQS model


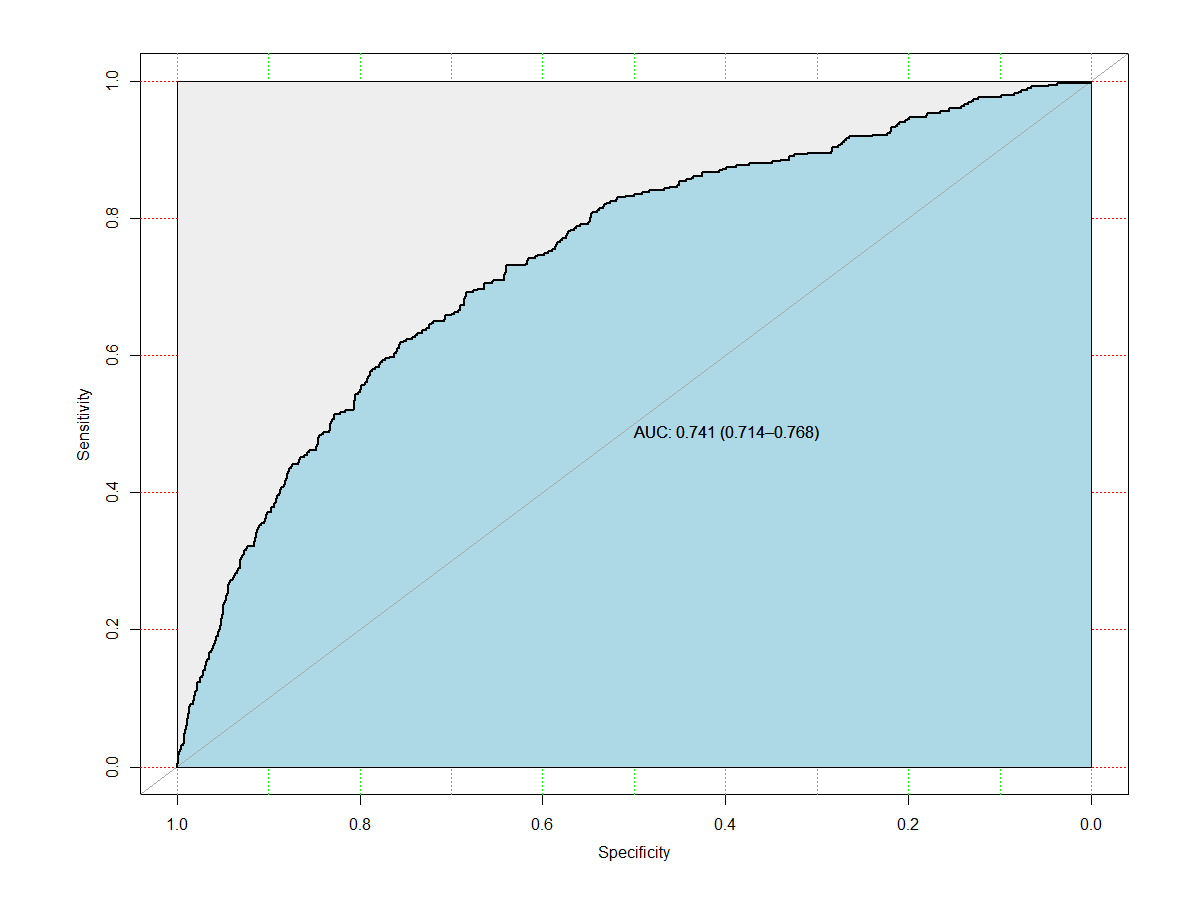


AUC, area under curve.
